# Advances in Laser Processing Technology of Materials

**DOI:** 10.3390/ma19040676

**Published:** 2026-02-10

**Authors:** Raffaele De Palo, Annalisa Volpe

**Affiliations:** 1 Department of Physics, Polytechnic University of Bari, Via G. Amendola 173, 70125 Bari, Italy; raffaele.depalo@poliba.it; 2 National Research Council (CNR), Institute for Photonics and Nanotechnologies (IFN), Via G. Amendola 173, 70125 Bari, Italy

Laser–material interaction processes have become a cornerstone of modern material engineering, bridging the gap between fundamental understanding and technological innovation. Laser-based techniques today are widely employed not only for conventional metals and alloys [1,2] but also for advanced materials such as ceramics, dielectrics, composites, and biomaterials [3,4,5,6,7].

In recent years, remarkable progress has been driven by two key factors: the development of novel laser sources [8,9]—including ultrashort pulses, high-power fiber lasers, and extended wavelength ranges—and the growing integration of numerical modeling, multiphysics simulation, and artificial intelligence into process design and control [10,11]. This synergy is enabling precise tailoring of surface morphology, structure, and functionality at the micro- and nanoscale, while reducing the thermal impact on the material [12].

Laser processing technologies and laser-based approaches play an increasingly important role across a wide range of scientific and technological fields. Thanks to their intrinsic versatility, lasers are now widely employed in micro- and nanofabrication, additive manufacturing, surface functionalization, and property tailoring, enabling controlled modification of optical, wetting, and tribological behavior. Beyond traditional manufacturing, laser technologies have also found growing applications in the biomedical field [13,14], where their precision and minimal invasiveness allow for advanced surface treatments, microstructuring, and functionalization of biocompatible materials [15].

In this context, this Special Issue will collect high-quality contributions that address both the fundamental aspects of laser–matter interaction—including the underlying physics, chemistry, and mechanics—and emerging applications of laser processing in materials science and engineering. The contributions span topics ranging from micro- and nanofabrication to additive manufacturing, surface engineering, and functional property modification, providing a comprehensive overview of recent advances in laser processing technology.

The papers in this Special Issue illustrate the remarkable versatility of laser processing technologies in shaping material morphology, structure, and properties. A recurring theme is the precise control of laser parameters to optimize performance and functionality across diverse material classes.

Several contributions focus on surface functionalization and microstructuring. Sun et al. fabricated superhydrophobic ultra-fine brass wires through laser texturing, demonstrating a scalable route to achieve strong water repellency on curved metallic geometries [16]. Atanasov et al. applied nanosecond laser processing to AlN ceramics to produce Al nanostructures for Surface-Enhanced Raman Spectroscopy (SERS), highlighting the potential of laser-induced surface morphology control in sensing applications [17].

The theme of laser–dielectric interaction is represented by Barbato et al., who developed nanochannels in fused silica via NaOH etching assisted by femtosecond laser irradiation, revealing unprecedented etching selectivity and precision [18]. This work exemplifies the growing importance of hybrid laser–chemical methods for fabricating 3D microfluidic architectures.

In the context of process optimization and intelligent manufacturing, Ružiak et al. combined artificial neural networks (ANNs) with experimental data to predict the influence of CO_2_ laser parameters on the kerf geometry of spruce wood, showing how AI can improve parameter selection for organic materials [19]. The predictive modeling approach represents a clear step toward data-driven control of laser processing.

Laser processing for metal cutting and forming is discussed by Paksoy et al., who introduced a water-jet-guided laser cutting system for AISI 1020 steel, achieving enhanced surface quality compared to conventional dry cutting [20]. This hybrid system expands the operational window of laser machining by combining optical guidance and thermal moderation.

The additive manufacturing and deposition domain is advanced by Yan et al., who analyzed the effect of scanning strategy on thermal behavior and residual stress in damping alloys during selective laser melting (SLM), revealing the crucial link between scanning trajectory, stress distribution, and mechanical stability [21]. Complementarily, Lorusso et al. explored spot size control in ultrashort laser deposition, demonstrating how focal conditions govern film thickness and uniformity in femtosecond-driven deposition processes [22].

A distinct contribution to energy materials is provided by Sikora et al., who reported on femtosecond-laser burst-mode structuring of Li-ion battery electrodes. Their work shows how burst-mode irradiation enhances active surface area and electrolyte accessibility, leading to improved electrochemical performance [23].

Two comprehensive review papers enrich the collection. He et al. present a detailed overview of metal material processing using femtosecond lasers, discussing the theoretical foundations, mechanisms, and broad applications of ultrafast laser technology in micromachining and biomedical contexts [24]. Perrone et al. offer an extensive review on laser cleaning and its influence on the quantum efficiency of metallic photocathodes, highlighting how laser-based surface treatments can improve photoinjector performance for accelerator technologies [25].

As Guest Editors, we are confident that the works included in this Special Issue will not only advance our understanding of laser–material interactions but also inspire future developments in functional materials, surface engineering, and next-generation manufacturing technologies.

We sincerely thank all authors and reviewers for their valuable contributions and the editorial team at Materials for their continuous support.
